# Effects of health education on adolescents’ non-cognitive skills, life satisfaction and aspirations, and health-related quality of life: A cluster-randomized controlled trial in Vietnam

**DOI:** 10.1371/journal.pone.0259000

**Published:** 2021-12-01

**Authors:** Sangchul Yoon, Shinki An, Dave Haeyun Noh, Le Thanh Tuan, Jongwook Lee

**Affiliations:** 1 Department of Medical Humanities and Social Sciences, College of Medicine, Yonsei University, Seoul, Republic of Korea; 2 Department of Ophthalmology, College of Medicine, Yonsei University, Seoul, Republic of Korea; 3 Department of Medical Education, College of Medicine, Yonsei University, Seoul, Republic of Korea; 4 Department of Agricultural and Resource Economics, University of Maryland, College Park, United States of America; 5 Department of Quality Testing, Thanh Hoa Medical College, Thanh Hoa, Vietnam; 6 Department of Agricultural Economics and Rural Development, College of Agriculture and Life Sciences, Seoul National University, Seoul, Republic of Korea; Washington University in St. Louis, UNITED STATES

## Abstract

**Objective:**

The effectiveness of health education on adolescents has been questioned, along with a growing body of empirical studies documenting the absence of behavioral changes after the intervention. However, evidence on its impact on other crucial health domains, besides health practices, is lacking. We evaluated the causal effects of a school-based health education program on adolescents’ multidimensional psychological health factors.

**Design:**

A cluster-randomized controlled trial.

**Methods:**

We conducted a cluster-randomized controlled trial involving 140 lower secondary schools in Vietnam. After stratifying by district, schools were randomized 1:1 to either treatment or control groups. Students enrolled in the treatment schools received monthly stand-alone health education in five topics by school teachers at the class level, but control group students did not receive any intervention. The primary outcomes of the study were students’ non-cognitive skills, life satisfaction, aspirations gap, and the Health-Related Quality of Life at five-month follow-up. We estimated the intention-to-treat effects with the panel fixed effects model using student panel data.

**Results:**

Of the 6,477 students enrolled at baseline, 2,958 (92%) treated and 2,967 (91%) control students completed the follow-up survey five months after baseline data collection from October to December 2018. Compared with controls, health education led to improved treatment school students’ self-efficacy (p-value = 0.013), presumed life satisfaction five years from the present (p-value = 0.001), aspirations gap for a socially and mentally healthy future (p-value = 0.036), and the Health-Related Quality of Life (p-value = 0.036).

**Conclusion:**

A school-based health education program enhanced students’ non-cognitive skills, life satisfaction and aspirations gap, and the Health-Related Quality of Life significantly. This study proposes essential psychological factors that should be taken into account when evaluating the effectiveness of a health education program in resource-limited settings.

## Introduction

Adolescents are a vulnerable group in public health, along with rapid physical and emotional changes resulting from increased hormones and social context changes during puberty. Transitioning from primary to secondary schools, the chance of engaging in risky health behaviors surges substantially as teenagers encounter older students with distinct group norms and peer pressure. However, adolescents often initiate risky behaviors without knowing potential consequences of such actions. Most of the teenagers’ sexual activities are unprotected [1], while sexual debut during adolescence [2] and an increasing proportion of the young population experiencing premarital sex [3] are widely reported. The majority of the cigarette smoking population starts smoking from adolescence [4], when peer pressure plays a significant role. Besides, teenagers tend to show low adherence levels in exercising preventive measures, such as handwashing [5] and physical activities [6] to avoid infectious diseases, myopia, and obesity.

While adolescence is a critical stage of the life cycle that requires social protection, suboptimal levels of attention and care are often provided in Low- and Middle-Income Countries (LMICs) due to limited resources, cultural barriers, and the policymakers’ lack of interest. For example, uncorrected refractive errors among adolescents are a primary cause of vision impairment in Vietnam, where about 20 percent of lower secondary school students are estimated to have myopia [7]. Despite increasing abortion rates, Vietnamese adolescents are excluded from the national population policy, leading to approximately 20 percent of abortions involving teens [8]. Competitive environments in school leave little room for adolescents to spend time on outdoor activities in the middle-income country, where more than 85 percent of teenagers do not spend sufficient time on physical activities [6].

The cost of risky health behaviors is substantial for both individuals and societies. Engaging in unprotected sex at a young age may lead to Sexually Transmitted Infections (STIs) [9], mental health problems [10], or unplanned pregnancies [11], causing severe health outcomes to teenagers. In particular, unwanted pregnancies of teenage girls are an urgent global challenge given its subsequent problems, such as complications during pregnancy and delivery [12], unsafe abortions [1], interrupted schooling [13], and loss of future earnings [14]. Likewise, smoking in adolescence may incur severe health issues—respiratory illnesses [15], interruption in brain development [16], and impairment in working memory [17]—which leads to limited job opportunities in the future [18]. The short-run and long-run adverse effects of risky behaviors are reported in preventive health domains, namely not washing hands at critical times [19] and not engaging in outdoor physical activities [6, 7]. Besides, risky health behaviors of teenagers increase the burden on societies by escalating health care costs while losing human capital. A significant amount of taxpayers’ money is spent on social problems attributed to unintended pregnancies [20], more than five percent of global health expenditure goes to smoking-related healthcare costs [21], and malnutrition-related healthcare claims up to USD 3.5 trillion per year globally [22]. Human capital loss caused by risky health behaviors is considerable. Apart from approximately 20 percent and 11 percent of global deaths resulting from communicable, maternal, neonatal, and nutritional (CMNN) diseases [23] and smoking [24], respectively, risky health behaviors deprive societies of human capital accumulation as adolescents become pregnant [25], smoke [16, 17], experience a vision problem [26], have an unbalanced diet [27], and suffer from an infectious disease [28].

Health education is a widely observed intervention designed to prevent such damaging health behaviors of teenagers in both LMICs and High-Income Countries (HICs). One of the underlying assumptions of health education is that an economic agent engages in unsafe health practices because her perceived immediate benefits are greater than the perceived future costs of such behaviors [29]. Hence, health education aims to prevent risky health behaviors by updating the agent’s perceived future costs resulting from the risk factors. The majority of the information-based programs target teenagers, when individuals’ health attitudes and behaviors are formed [30, 31]. In particular, schools are a vital place to implement a health education program to reach a large number of adolescents for years [32, 33] in a financially sustainable and logistically convenient way thanks to existing learning structures [34], ensuring the high returns to the intervention [35].

However, the effectiveness of health education has been questioned, along with a mounting body of empirical evidence documenting the null effects of the information-based approach in behavioral changes. Despite a few studies presenting significant effects [36, 37], the overwhelming consensus from existing empirical evidence is that a health education program may increase teenagers’ knowledge in health, but translating it into behavioral changes is exceptionally challenging. The findings are consistent across topics, including sexual and reproductive health [38], anti-smoking [32], eye health [39], hygiene [40], and nutrition [41], leading to the conclusion that cost-effectiveness of health education is an *‘illusion’* [42].

Although much of the literature focused on the effects of health education on teenagers’ Knowledge, Attitudes, and Practices (KAP) in health, there exists limited evidence on psychological factors related to adolescents’ current and future health outcomes, such as non-cognitive skills, the quality of life, and life satisfaction and aspirations gap. For example, self-efficacy, personal beliefs in own capacity [43], is an essential mediating component necessary when translating knowledge into action [44] as it shapes one’s behavioral intentions. A teenager’s life satisfaction elicits how healthy a student is physically, mentally, and socially, which are associated with school life [45], other risky health behaviors [46], social problems, and mental health in both positive [47] and negative [48] ways. Finally, the Health-Related Quality of Life (HRQoL), an individual’s self-perceived multidimensional health domains [49] beyond morbidity and mortality, captures both the self-assessed physical and mental health status of adolescents, serving as an indicator of current health and a predictor of future health outcomes [50].

In this study, we examined the effects of a health education program on non-cognitive skills, life satisfaction, aspirations gap, and HRQoL besides health KAP. We conducted a randomized-controlled trial in Vietnam to investigate the impacts of school-based health education on adolescents’ psychological health which should not be neglected when evaluating the information-based approach. Randomly selected lower secondary school students in Thanh Hoa province received monthly stand-alone health education in five topics: Eye Health; Sexual and Reproductive Health (SRH); Infectious Diseases and Handwashing; Food and Nutrition; and Anti-Smoking at the class level. Treated students learned essential health information and life skills necessary to make sound health decisions from trained school teachers. We assessed impacts of the health education program by comparing the treatment students to their control group counterparts five month after baseline data collection.

## Methods

### Study designs, randomization, and participants

**Fig 1:**
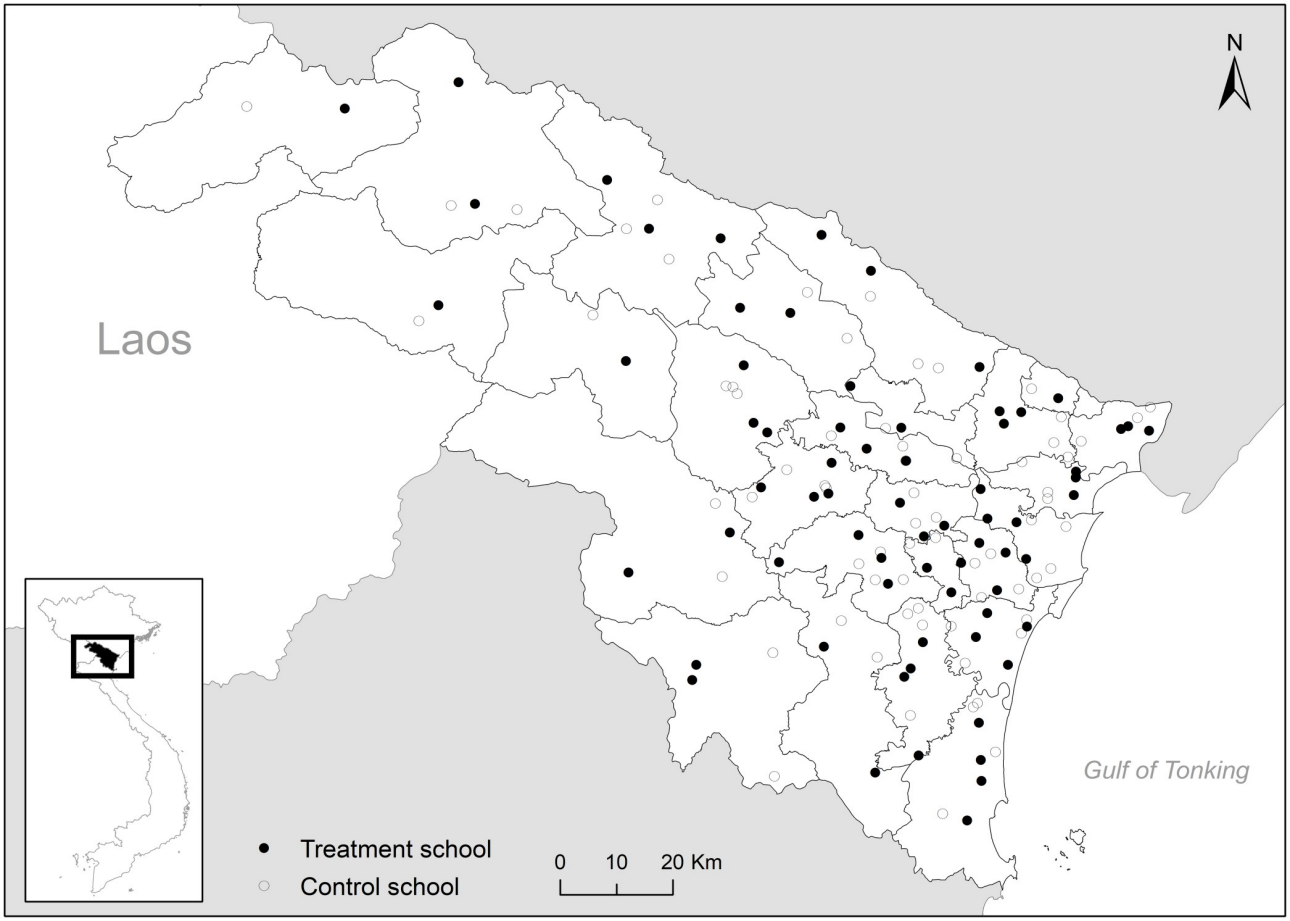
Study area. This figure plots study schools and district boundaries in Thanh Hoa province, Vietnam. The treatment schools are denoted by solid circles, while the control schools are denoted by hollow circles. Source: Government of Viet Nam.

We conducted a cluster-randomized controlled trial in lower secondary schools in Thanh Hoa province, Vietnam, from 2018 to 2019. From all of 652 public lower-secondary schools across the province, 140 schools were randomly selected based on the total number of lower-secondary schools in each district (Fig 1). The schools were assigned to either the treatment (70 schools) or the control (70 schools) groups after stratifying by the district. Randomization took place at the school level rather than the student level to take into account spillover effects and to minimize potential ethical issues. All the selected treatment and control schools agreed to participate in the program. We distributed two types of consent forms for students to take home—one about the health education program participation to all treatment school students and another about survey participation to a subset of treatment and control school students. Of these, students who returned the form signed by their parent or guardian were enrolled in the program and the study. All the participating students in the treatment group received a series of health education sessions, while none of these was provided to the control group students.

In each school, approximately 48 students were randomly sampled for surveys after stratifying by school class and sex of students. Inclusion criteria for the study were the randomly selected student cohorts in grades 6, 7, 8, and 9 in the 2018–2019 academic year (aged 11 to 14) whose caregivers gave consent for their children to participate in the surveys. Also, students were required to provide assent and be able to read and speak Vietnamese fluently to join the study. Our analytic sample includes those who completed both baseline and follow-up surveys. However, including students who were surveyed at follow-up regardless of baseline survey participation in the sample does not change results significantly. Exclusion criteria were students who refused assent; whose parents or guardians declined for their children to join the study; and those who were unable to speak or read Vietnamese. Students who had been transferred to other schools between baseline and follow-up surveys were also excluded from our analysis. Ethical approval for the study was obtained from the Yonsei University Institutional Review Board (IRB ID: 4–2018-1060) and the University of Minnesota Institutional Review Board (IRB ID: STUDY00004327).

### Procedures

Once a month, trained teachers instructed a 45-minute-long health education session in the treatment schools at the class level as a stand-alone course over five months. A total of five health topics, namely Eye Health, SRH, Infectious Diseases and Handwashing, Food and Nutrition, and Anti-Smoking, took place sequentially on regular school days. Each session consisted of two parts—lectures and in-class activities. A teacher started each session by explaining what constitutes risky health behaviors, why they matter, and how to prevent them (i.e., lecture), followed by student-centered participative activities (i.e., in-class activities) when students learned essential life skills to protect their own health from damaging health behaviors.

The health program aimed to reduce risky health behaviors of participating adolescents, including unprotected sex, sugar overconsumption, and cigarette smoking, and to increase their adherence to preventive health practices, namely outdoor activities and washing hands at critical times. The program was designed to enhance students’ health knowledge and attitudes by updating their perceived future costs associated with such behaviors. Given the improved understanding and perspectives in risky behaviors, life skills acquired from in-class activities were to enable them to avoid risky behaviors. Along the way of obtaining crucial information and life skills, health education was expected to promote students’ non-cognitive skills as their beliefs in personal ability to control their own behaviors improve, leading to increased life satisfaction and HRQoL.

Before providing health education to students, we trained treatment school teachers to serve as health education instructors via the Training of Trainers. Two health teachers from each treatment school recruited by headmasters were invited to two-day training sessions. Using the teaching guidelines approved by the Department of Education and Training, professors at the Thanh Hoa Medical College led the training sessions. During the training sessions, the teachers learned what to teach (i.e., health promotion messages) and how to teach (i.e., pedagogical skills) using the guidelines. After completing training, the health teachers had organized another workshop at the school level, serving as peer educators for homeroom teachers who delivered health promotion messages to students at the class level. The homeroom teachers taught all health topics except SRH, which health teachers instructed, given the sensitivity of the topic. Pre- and post-training evaluation reveals that the trained teachers had a better understanding of teaching materials after completing training.

We collected a rich array of data, such as students’ demographic information (e.g., age, sex, ethnicity, mother tongue, and the number of household members living together), school life, health KAP in five topics, non-cognitive skills, life satisfaction, aspirations gap, and HRQoL from in-person surveys. Moreover, students’ health information such as height, weight, chest circumference, vision acuity, hearing ability, blood pressure levels, and dental problems, was collected from the treatment school students immediately after the baseline survey. The follow-up survey took place from March to April 2019, approximately five months after baseline data collection from October to December 2018, but the second follow-up survey scheduled to be collected in 2020 was interrupted due to the COVID-19 pandemic.

### Outcomes

We measured outcomes at the individual level. The primary outcomes of the study were non-cognitive skills (i.e., self-esteem and self-efficacy), life satisfaction and aspirations gap, and HRQoL, and the secondary outcomes were health knowledge and practices in the five topics students had learned. We calibrated levels of life satisfaction and aspirations gap by using an adapted version of the Cantril Ladder [51], where respondents were asked to indicate where they thought that they were at the present time and five years from the present using a zero (the worst) to nine (the best) scale. Students’ aspirations gap was computed as the difference between the expected future life satisfaction and the current life satisfaction. The KINDL-R questionnaire [52] was used to measure HRQoL such as students’ physical well-being, emotional well-being, self-esteem, family, friends, and school life. Students’ health knowledge scores were constructed by using the two-parameter logistic Item Response Theory model [53], and we used students’ self-reported answers for health practice outcomes in our analysis. For sensitive health practice questions such as sexual intercourse and smoking, students had an option to choose *“I do not know,”* which was coded as missing.

### Statistical analysis

The unit of analysis was an individual student. We estimated the intention-to-treat (ITT) effects where the impacts of offering the health program were evaluated regardless of compliance. First, we assessed whether the baseline characteristics of the treatment and control groups were statistically different. We then examined the treatment effects of health education by the panel fixed effects model, since students who had been surveyed at baseline were visited again for the follow-up survey. Continuous outcome variables were normalized by the means and standard deviations of the control group values of corresponding variables measured at baseline to report standardized effect sizes. Theoretically, the randomization allows us to estimate unbiased treatment effects without covariates. However, we included some key individual characteristics—students’ age, the number of siblings, and the number of rooms per household member—as control variables in addition to the student fixed effects, mainly due to baseline imbalances between the treatment and control groups to increase precision. Time-invariant characteristics, such as ethnicity and locality of schools, were excluded from the vector of covariates because the student fixed effects control for any differences attributed to factors that do not change across time. Throughout the analysis, standard errors were clustered at the school level—the unit of randomization. We used Stata version 15.1 for statistical analysis with 5 percent statistical significance level criteria.

## Results

**Fig 2:**
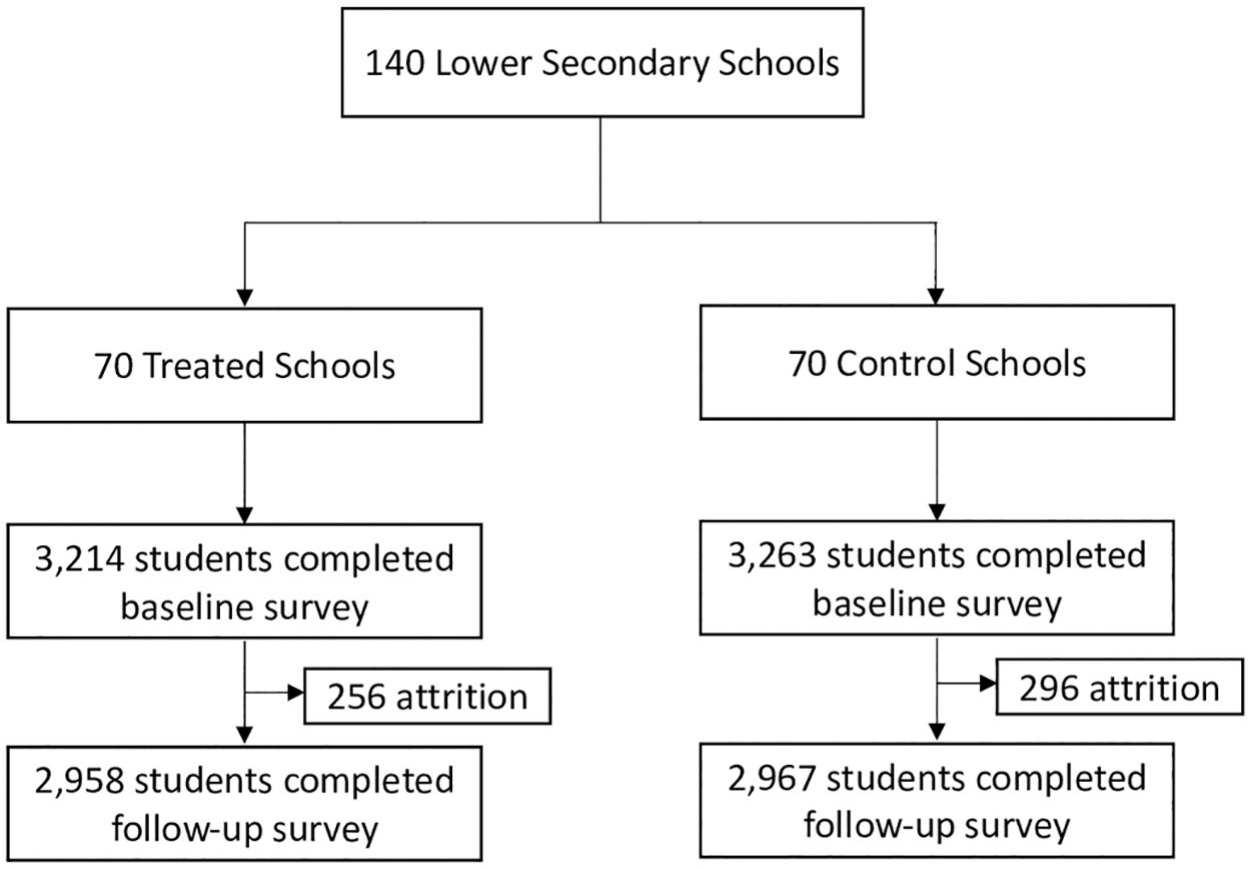
Trial profile. The follow-up survey took place approximately five months after the baseline survey. Students who had been surveyed both at baseline and follow-up were included in the sample.

Details of the study sample are demonstrated in Fig 2. Of the 6,477 enrolled students at baseline, 5,925 students (2,958 treated and 2,967 control students) who had completed an assessment at five months were included in the study. The attrition rates of the treatment and control groups were eight percent and nine percent, respectively, but the differences in the baseline characteristics of the lost students to follow-up were marginal, and they were not statistically significant at the 5 percent level.

Table 1 presents baseline characteristics of the study participants by treatment status. Panel A confirms that the two groups were balanced, on average, except for a few variables with small differences in magnitude given a large sample size. About half of the respondents were female, and the average age of participants was between 12 and 13 years, reflecting the random sampling stratified by gender and class. The average household size was 4.7 for both groups, and 51 percent of respondents were the first child of the families. More than 97 percent of students in our study sample had answered that they had lived with at least one of their parents, and we did not find any statistical difference between the treatment and control groups. Despite the random assignment of schools, we found four demographic variables—ethnicity, language, the number of siblings, and the number of rooms per person—that were statistically different across groups. While the magnitude of the differences was small, we controlled for the number of siblings and the number of rooms per person when evaluating the treatment effects to increase precision, but ethnicity and language were excluded because of the student fixed effects that partial out any effects of time-invariant variables. Panel B reports two school characteristics—school size and locality—across treatment conditions. The average school size was about 270 students, which was in line with the General Statistics Office of Vietnam statistics [54] as a result of the random sampling of schools. The treated schools were more likely to be located in rural areas, but the difference was small, and the fixed effects model addresses any time-invariant factors, including locality.

**Table 1:** Student and school characteristics.

	Treatment		Control		t-test
	(N = 2,958)		(N = 2,967)		(N = 5,925)
	Mean	SD	Mean	SD	p-value
**Panel A: Demographic Characteristics**					
Female (0–1)	0.51	0.50	0.52	0.50	0.351
Age (Years)	12.79	1.19	12.76	1.20	0.321
Ethnicity: Kinh (0–1)	0.75	0.43	0.79	0.41	<0.001
Language: Vietnamese (0–1)	0.77	0.42	0.82	0.38	<0.001
First Child (0–1)	0.52	0.50	0.51	0.50	0.683
Number of Household Members	4.75	1.46	4.74	1.35	0.585
Number of Siblings	1.64	1.24	1.49	1.03	<0.001
Number of Rooms/person	0.53	0.31	0.55	0.30	0.003
Living with Both Parents (0–1)	0.89	0.31	0.88	0.32	0.436
Living with Other Guardians (0–1)	0.03	0.17	0.03	0.18	0.313
Living with Mother Only (0–1)	0.06	0.23	0.06	0.24	0.641
Living with Father Only (0–1)	0.02	0.16	0.02	0.15	0.810
**Panel B: School Characteristics**					
School Size (Number of Students)	270.99	120.40	282.53	116.69	0.520
Rural (0–1)	0.86	0.35	0.77	0.42	0.171

Note: The sample includes students who participated in both the baseline and the follow-up surveys. The p-values from the t-test of the null hypothesis that H_0_ : *β*_1_ = 0 in the regression Variable = *β*_0_ + *β*_1_ × Treat + DistrictDummies + *ϵ* are reported as randomization took place at the district level.

We also conducted balance tests for both primary and secondary outcome variables. While Table 2 shows that some variables are statistically different across groups given a large number of observations, magnitudes are small, and the student fixed effects take into account any time-invariant pre-treatment differences.

**Table 2:** Balance test for dependent variables.

	Treatment		Control		t-test
	(N = 2,958)		(N = 2,967)		(N = 5,925)
	Mean	SD	Mean	SD	p-value
**Panel A: Non-cognitive Skills**					
Self-Esteem (0–100)	70.77	18.82	69.39	18.66	0.004
Self-Efficacy (0–100)	69.17	13.92	69.93	13.72	0.019
**Panel B: Life Satisfaction**					
Present (1–9)	6.54	1.64	6.46	1.61	0.062
Future (1–9)	7.43	1.44	7.52	1.39	0.010
Aspirations gap (Future-Present)	0.89	1.56	1.06	1.55	<0.001
**Panel C: Health-Related Quality of Life**					
Aggregated (0–100)	69.23	10.67	68.97	10.86	0.420
Physical Well-being (0–100)	72.48	14.97	73.70	15.52	0.001
Emotional Well-being (0–100)	74.17	15.16	73.68	15.61	0.293
Self-esteem (0–100)	54.77	20.75	53.55	20.12	0.020
Family (0–100)	80.67	15.15	81.05	14.91	0.279
Friends (0–100)	74.31	16.24	73.81	16.54	0.320
School (0–100)	58.96	16.81	58.00	16.96	0.025
**Panel D: Health Knowledge**					
Aggregated (0–100)	62.48	9.25	62.74	8.36	0.224
Eye (0–100)	57.91	15.96	57.21	14.99	0.100
SRH (0–100)	55.07	15.26	54.37	14.15	0.067
Handwashing (0–100)	77.97	15.81	79.94	15.42	<0.001
Food & Nutrition (0–100)	42.88	13.74	42.93	13.46	0.910
Anti-Smoking (0–100)	78.55	16.04	79.23	15.07	0.083
**Panel E: Health Practices**					
Outdoor Activities (Likert, 1–5)	3.41	1.03	3.40	1.02	0.675
Had Sex (0–1)	0.04	0.21	0.02	0.15	<0.001
Handwashing, Eating (0–1)	0.96	0.20	0.97	0.17	0.002
Handwashing with Soap, Eating (0–1)	0.88	0.33	0.86	0.34	0.225
Handwashing, Toilet (0–1)	0.96	0.21	0.97	0.17	0.004
Handwashing with Soap, Toilet (0–1)	0.91	0.29	0.91	0.29	0.570
Snacks (Likert, 0–5)	3.89	1.41	3.80	1.43	0.018
Had Smoked (0–1)	0.04	0.20	0.03	0.17	0.044

Note: The sample includes students who participated in both the baseline and the follow-up surveys. The p-values from the t-test of the null hypothesis that H_0_ : *β*_1_ = 0 in the regression Variable = *β*_0_ + *β*_1_ × Treat + DistrictDummies + *ϵ* are reported as randomization took place at the district level.

**Fig 3:**
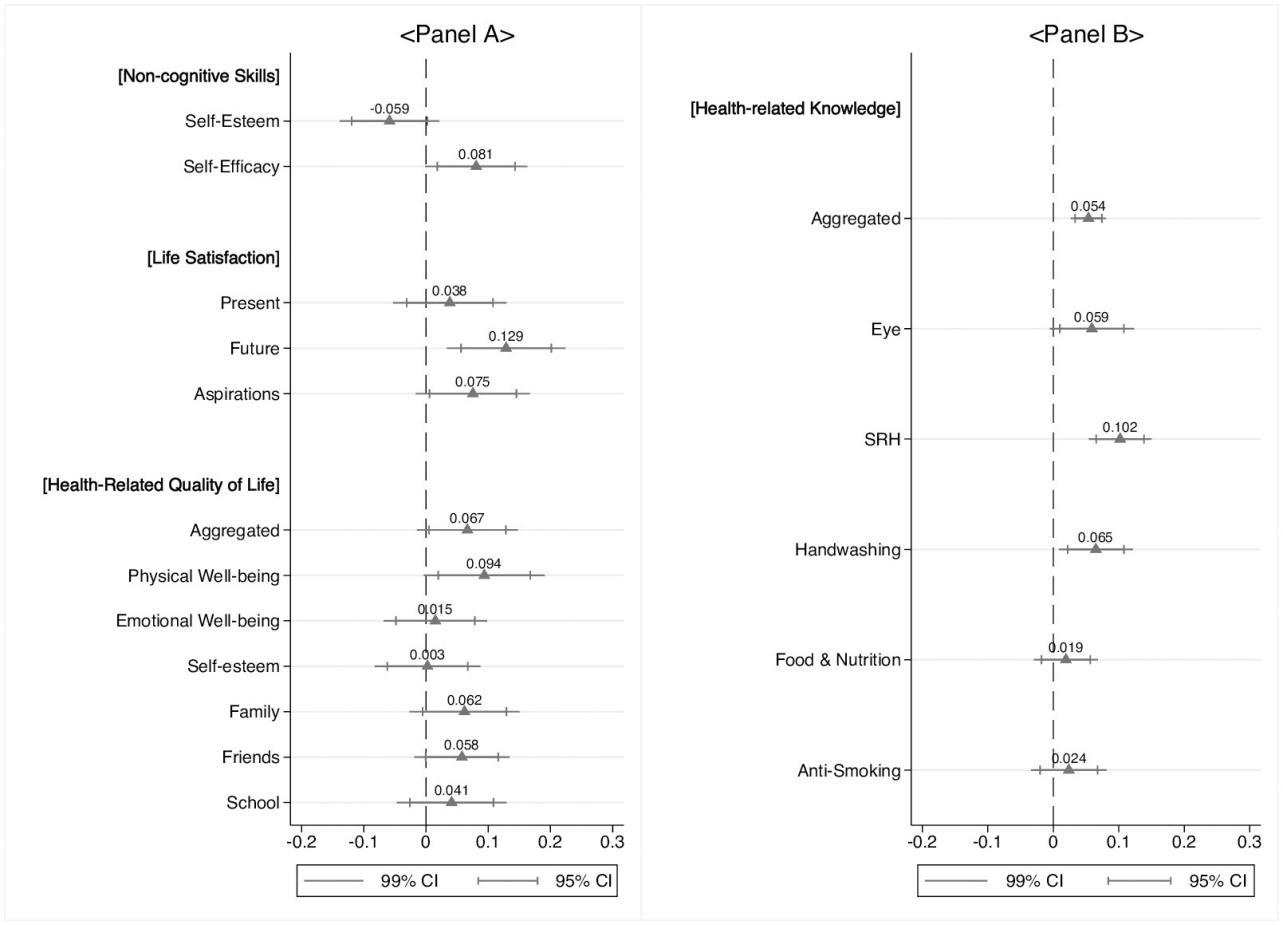
Treatment effects. Coefficients and confident intervals estimated from the panel fixed effects model are plotted. Standard errors were clustered at the school level. Students’ age, the number of siblings, and the number of rooms per household member were included as control variables in addition to the student fixed effects. Continuous outcome variables were normalized by the means and standard deviations of the control group values of corresponding variables measured at baseline. Students’ knowledge levels were constructed by using the two-parameter logistic IRT model. Students who had been surveyed both at baseline and follow-up were included in the sample.

Panel A of Fig 3 summarizes the treatment effects on students’ non-cognitive skills, life satisfaction and aspirations gap, and HRQoL. Despite the insignificant effects on self-esteem, we found that the health education program increased students’ perceived beliefs in their own capacity by 0.081 SDs (p-value = 0.013). Besides, students’ life satisfaction increased substantially after receiving health education on five topics. The current life satisfaction of students was 0.038 SDs (p-value = 0.281) higher in the treatment group than the control group, but it was not statistically significant at the 5 percent level. However, when the students were asked where they thought they would stand five years from the present, the treated students’ expectation regarding their future was increased by 0.129 SDs (p-value = 0.001) compared to the control group, leading to the 0.075 SDs (p-value = 0.036) higher aspirations gap after receiving health education. We then assessed the effects of the school-based health education program on students’ HRQoL. Overall, we found positive treatment effects on all aspects of HRQoL. While physical well-being was the only HRQoL sub-component that had a significant treatment effect, we found positive coefficients for all the other sub-components, leading to 0.067 SDs (p-value = 0.036) higher aggregated HRQoL scores from the treatment school students than the control group.

We also investigated how the school-based health program had affected primary outcomes of the existing health education literature: students’ health knowledge and practices. Findings from this study are consistent with a rapidly growing body of empirical evidence: significant effects of health education on adolescents’ knowledge but limited effects on behavioral changes. Overall, students’ health knowledge increased significantly after receiving monthly health education (Panel B of Fig 3). Student’s aggregated health-related knowledge increased by 0.054 SDs (p-value<0.001) mostly attributed to the effects on Eye Health (*β*=0.059, p-value = 0.020), SRH (*β*=0.102, p-value<0.001), and Infectious Diseases and Handwashing (*β*=0.065, p-value = 0.004). While we found higher knowledge scores in Food and Nutrition (*β*=0.019, p-value = 0.313) and Anti-smoking (*β*=0.024, p-value = 0.291) from the treated students relative to the control group counterparts, the differences are not statistically significant.

Table 3 shows mixed results for behavioral changes despite the positive treatment effects on students’ knowledge in health. While students reduced risky behaviors in two areas, Infectious Diseases and Handwashing and Food and Nutrition, they did not change health behaviors in Eye Health, SRH, and Anti-smoking. First, students’ handwashing behaviors improved significantly after receiving health education (Columns 3–6). The percentages of teenagers who answered that they wash their hands before eating a meal (*β*=0.019, p-value = 0.008) and after using the toilet (*β*=0.018, p-value = 0.010) with or without soap increased significantly, while the effects on handwashing behaviors with soap were not significant. The null effects on handwashing with soap are consistent with existing studies concluding that improvement in handwashing with soap behaviors requires the provision of soap. Column 7 shows that health education led to significantly decreased sugar consumptions from snacks and soft drinks among adolescents (*β*=-0.073, p-value = 0.014). However, estimates for the other health behaviors were noisy. After receiving health education, students were more likely to engage in regular outdoor activities at least one hour per day as a preventive measure of myopia (Column 1), but the group difference between the treatment and control students was not significant at the 5 percent level (p-value = 0.224). Finally, we found insignificant program effects on students’ initiation of sexual activity (p-value = 0.517) and smoking (p-value = 0.153) as reported in Columns 2 and 8, respectively.

**Table 3:** Health-related practices.

	Eye	SRH	Handwashing				Food	Anti-Smoking
	(1)	(2)	(3)	(4)	(5)	(6)	(7)	(8)
	Outdoor	Sex	Eating	Eating (Soap)	Toilet	Toilet (Soap)	Snacks	Smoked
Treat	0.042 (0.034)	0.002 (0.003)	0.019** (0.007)	-0.001 (0.012)	0.018* (0.007)	-0.004 (0.011)	-0.073* (0.030)	-0.007 (0.005)
Mean	3.396	0.022	0.971	0.865	0.970	0.906	3.799	0.031
SD	1.023	0.145	0.167	0.342	0.171	0.292	1.431	0.174
FE	X	X	X	X	X	X	X	X
Controls	X	X	X	X	X	X	X	X
R^2^	0.0027	0.0110	0.0016	0.0003	0.0016	0.0004	0.0042	0.0309
N	11,848	10,694	11,848	11,848	11,848	11,848	11,848	11,506

Note:

*P < 0.05,

**P < 0.01,

***P < 0.001.

Standard errors in parentheses. The panel fixed effects model was used to estimate the treatment effects. Standard errors were clustered at the school level. Students’ age, the number of siblings, and the number of rooms per household member were included as control variables in addition to the student fixed effects. Continuous outcome variables were normalized by the means and standard deviations of the control group values of corresponding variables measured at baseline. Students who had been surveyed both at baseline and follow-up were included in the sample.

Table 4 reports heterogeneous treatment effects across gender and age. Panel A shows that, on average, male students benefited from the program more than female counterparts. First, male students’ aspirations gap increased significantly by 0.182 SDs, while the effects on female students are not significant, leading to a statistically significant difference across gender by 0.105 SDs. The differential effects of the program on students across gender are well-manifested in HRQoL outcomes from which the group differences are observed for both aggregated index and sub-components, namely physical well-being, emotional well-being, self-esteem, and school life. The table shows that receiving health education has no significant effects on females students’ most of the HRQoL outcomes, but it has positive effects on male counterparts for all of HRQoL variables except family, leading to heterogeneous treatment effects across gender within the treatment group. Panel B shows that the effects of the health education program were larger for younger students. The treatment effects on the aggregated HRQoL decreased by 0.057 SDs when a student was one year older, and we found similar results from sub-components.

**Table 4:** Heterogeneity.

	Treat × Group		Treat		Treat × Group+Treat		N
	Coef.	SEs	Coef.	SEs	Coef.	SEs	
**Panel A: Female**							
*Non-cognitive Skills*							
Self-Esteem	-0.039	0.045	-0.039	0.038	-0.078*	0.039	11,850
Self-Efficacy	-0.028	0.048	0.095*	0.044	0.067	0.035	11,850
*Life Satisfaction*							
Present	-0.000	0.046	0.038	0.040	0.038	0.044	11,850
Future	-0.105*	0.045	0.182***	0.043	0.077	0.043	11,850
Aspirations gap	-0.093	0.055	0.123**	0.045	0.030	0.046	11,850
*HRQoL*							
Aggregated	-0.188***	0.034	0.162***	0.033	-0.026	0.038	11,850
Physical Well-being	-0.211***	0.046	0.201***	0.045	-0.010	0.043	11,850
Emotional Well-being	-0.131**	0.041	0.082*	0.036	-0.049	0.040	11,850
Self-esteem	-0.190***	0.038	0.099*	0.040	-0.091*	0.036	11,850
Family	-0.022	0.039	0.073	0.041	0.051	0.038	11,850
Friends	-0.037	0.043	0.077*	0.036	0.040	0.038	11,850
School	-0.124**	0.040	0.104**	0.039	-0.020	0.040	11,850
**Panel B: Age**							
*Non-cognitive Skills*							
Self-Esteem	0.030	0.019	-0.456	0.254			11,850
Self-Efficacy	-0.021	0.020	0.354	0.256			11,850
*Life Satisfaction*							
Present	0.018	0.019	-0.193	0.251			11,850
Future	0.021	0.019	-0.148	0.259			11,850
Aspirations gap	0.002	0.021	0.050	0.281			11,850
*HRQoL*							
Aggregated	-0.057***	0.014	0.815***	0.189			11,850
Physical Well-being	-0.042*	0.020	0.647*	0.266			11,850
Emotional Well-being	-0.067***	0.016	0.903***	0.218			11,850
Self-esteem	0.024	0.016	-0.307	0.214			11,850
Family	-0.024	0.017	0.382	0.233			11,850
Friends	-0.050**	0.015	0.723***	0.210			11,850
School	-0.078***	0.019	1.074***	0.255			11,850

Note:

*P < 0.05,

**P < 0.01,

***P < 0.001.

Standard errors in parentheses. The panel fixed effects model was used to estimate the treatment effects. Standard errors were clustered at the school level. Students’ age, the number of siblings, and the number of rooms per household member were included as control variables in addition to the student fixed effects. Continuous outcome variables were normalized by the means and standard deviations of the control group values of corresponding variables measured at baseline. Students who had been surveyed both at baseline and follow-up were included in the sample.

## Discussion

In this study, we focused on psychological health dimensions in addition to direct health knowledge and practices of adolescents as a result of health education. We reported that a school-based health education program had led to increases in adolescents’ self-efficacy, life satisfaction, aspirations gap, and HRQoL. However, the program had limited effects on reducing risky health behaviors—the primary objective of the information-based intervention.

First, our findings showed significant improvement in self-efficacy after receiving a series of health education classes. Combining with limited behavioral changes despite improved health knowledge, this is an important finding from the perspective of behavioral changes. There exist three potential mechanisms through which enhanced health knowledge and self-efficacy were not translated into reduced risky health behaviors. According to the theory of planned behaviors [55], the absence of behavioral changes is caused by students’ lack of intentions to adhere to lessons from health education. In other words, students who received health education had a better understanding of risky health behaviors (i.e., increased knowledge in health), believed that they had the capability to refrain from them (i.e., increased self-efficacy), but they might not have enough intentions or motivations to exercise their efforts, leading to the limited behaviors changes. On the other hand, the limited effects on health practices may reflect circumstantial factors that prevent teenagers from improving preventive measures while avoiding risky behaviors regardless of their intentions. The prototype willingness model [56] from the health psychology literature suggests that adolescents’ intentions play a limited role in health practices because their risky behaviors are more likely to be reactive to risk conducive environments rather than planned actions. For example, even if a student has strong intentions to spend more time on outdoor activities to prevent myopia and obesity, it may require parents and teachers’ permission whose one of the top priorities is academic success in school. Also, a student may be unable to wash their hands with soap despite the enhanced knowledge and attitudes simply because there exists no soap available at home and school as documented in the existing literature. [57]. Although including parents and teachers does not necessarily lead to successful behavior changes of teenagers [58], ensuring conditions under which a teen has an option to make a health decision is a prerequisite condition for a health program to have an impact on adolescents’ health behaviors. Finally, evaluating the treatment effects five months after baseline data collection may not give sufficient time for students to alter health behaviors, in particular in sexual intercourse and smoking, given low baseline prevalence. Despite serious problems caused by the risky behaviors during adolescence, less than four percent of students answered at baseline that they had had sex or had smoked before. Hence, examining the long-run effects is necessary before concluding that health education has no impact on behavior changes since risks of initiating such health behaviors increase substantially as a teen advances from lower secondary to higher secondary schools.

Secondly, this study showed the positive impacts of health education on critical health domains that received relatively limited attention in the health education literature: life satisfaction and aspirations gap. A growing strand of literature pays attention to teenagers’ subjective well-being given its strong association with behavioral [59, 60], social life [45, 61], and psychological [47, 61] problems of the core risk group. In particular, life satisfaction is a useful indicator of severe mental health issues, such as depression [62], loneliness [48], and suicide [63]. The significant effects on life satisfaction we found highlight the possibility of the information-based intervention to address mental health problems of adolescents by increasing their hope and aspirations gap for a socially and mentally healthy future.

Finally, we found that students’ perceived health, particularly in physical well-being, had improved significantly after participating in the school-based health education program. Despite the importance of HRQoL in assessing adolescents’ current and future health [50], the result should be interpreted with caution. First, the increased physical well-being may reflect mere changes in adolescents’ subjective perceptions rather than improvement in physical health per se because HRQoL was designed to measure a respondent’s self-assessed health status. Second, the present study did not allow us to disentangle the effects on physical well-being attributed to health education from the effects caused by physical examination, since it took place in the treatment schools only. While students’ increased physical well-being may indicate improved physical health as a result of reduced health behaviors, we cannot exclude other channels through which health assessment affected students. For example, the physical examination might improve students’ health status by identifying health issues that they were at risk for, leading to enhanced health conditions at follow-up. At the same time, the physical examination could function as an awakening tool for teenagers to confirm how healthy they were given that most students learned that they did not have health problems in vision (85 percent), hearing (97 percent), and dental (70 percent). Thus, measuring students’ physical health status in the treatment schools may spur participating students into an active assessment of their own health, concluding that *‘I am healthy,’* which could be reflected by higher physical health well-being scores relative to the control group.

There exist several limitations in this study. First, as mentioned above, isolating the treatment effects of health education on students from the school-based health check-up was difficult given the study design. While the physical examination was conducted as a part of data collection, it may play a role in behavioral changes if it induces participants to change their attitudes on certain behaviors as shown in existing studies [64, 65]. Second, we examined short-run impacts of the school-based health education program only because additional data collection activities had been interrupted by the COVID-19 pandemic. A further study investigating the long-run effects is necessary to examine whether the health education program failed to achieve behavior changes, and to assess to which extent the treatment effects on psychological factors remain. Finally, our outcomes may be subject to potential social desirability bias, given that we relied on students’ self-reported answers [66].

## Conclusion

This study demonstrated significant treatment effects of a health education program on adolescents’ vital psychological health domains: self-efficacy, life satisfaction, aspirations gap, and HRQoL. Taken together with mixed results for health practices, findings on self-efficacy revealed the teenagers’ limited intentions or potential risk-conducive circumstances that may prevent adolescents from avoiding risky health behaviors, shedding light on the last mile to be addressed to incur behavioral changes among the risk group. This study also documented the positive effects of a school-based health education program on psychological health dimensions of adolescents that received relatively limited attention in the health education literature. Significant improvements in students’ life satisfaction, aspirations gap, and HRQoL highlighted the necessity of taking into account the broader health dimensions that should not be neglected when evaluating impacts and effectiveness of a health education program on teenagers in resource-limited settings in LMICs.

## Supporting information

S1 Protocol The study protocol. The study protocol is described.
(PDF)
