# Supplementary material for: Effects of health education on adolescents’ non-cognitive skills, life satisfaction and aspirations, and health-related quality of life: A cluster-randomized controlled trial in Vietnam
Source: PLoS One. 2021 Dec 1;16(12):e0259000. doi: 10.1371/journal.pone.0259000 (PMC8635366; doi:10.1371/journal.pone.0259000)
Supplement: S1 Protocol — The study protocol is described. (PDF) [file pone.0259000.s001.pdf]

The Study Protocol

Implementing Organization
This study was implemented by an international non-profit organization–Project BOM (Blindness Zero Movement), which is affiliated with Yonsei University College of Medicine in South Korea with funding from the Korean International Cooperation Agency (KOICA).

Randomization
The study utilized a randomized controlled trail (RCT) design to evaluate the causal effects of health education in lower secondary schools in Thanh Hoa Province, Vietnam. Randomization was conducted at the school level, and the unit of observation is students. Out of 140 schools chosen from all of 652 lower secondary schools in Thanh Hoa province, 70 schools were randomly selected for the intervention while the other 70 schools were selected as comparison school.

Introduction to the Program to Schools
For the selected 140 schools in the Thanh Hoa province, stakeholders and school headmasters were invited to a kick-off meeting to introduce the program. All schools agreed to join the program.

Training Sessions for Health Teachers
First, the project team provided training for school health teachers (two teachers from each school, on average) with school education materials. The training was led by health professionals from Thanh Hoa Medical College. During the training sessions, the teachers learned what to teach (i.e., health promotion messages) and how to teach (i.e., pedagogical skills) using the guidelines. Second, the trained health teachers had organized another workshops at the school level, serving as peer educators for homeroom teachers who delivered health promotion messages to students at the class level.

Consent
We distributed two types of consent forms for students to take home—one about the health education program participation to all treatment school students and another about survey participation to a subset of treatment and control school students. Of these, students who returned the form signed by their parent or guardian were enrolled in the program and the study.

Baseline Survey
Baseline survey was conducted at the student level in schools for students who provided assent. By visiting all 140 schools in the sample, the research team interviewed students in schools. All the information was collected privately and confidentially, and all students were informed that they could choose not to answer any questions or stop at any time without any penalty.

Health Check-up
After collecting baseline information from students in the sample, health checkup was conducted by health professionals from the public health centers and project staff to collect students' health information such as height, weight, chest circumference, vision acuity, hearing ability, blood

pressure levels, and dental problems. Tablet PCs were used during the check-up to store health information.

Health Education

Once a month, trained teachers instructed a health education session in the treatment schools at the class level as a stand-alone course. Total five topics of health education such as food and nutrition, infectious diseases and handwashing, tobacco prevention, sexual and reproductive health, and eye health were provided.

Follow-up Survey

Follow-up survey was conducted at the end of school year for students who were sampled for survey. Assent was obtained from students before answering any questions, and all students were informed that they could choose not to answer any questions or stop at any time without any penalty.
