# Supplementary material for: Effects of health education on adolescents’ non-cognitive skills, life satisfaction and aspirations, and health-related quality of life: A cluster-randomized controlled trial in Vietnam
Source: PLoS One. 2021 Dec 1;16(12):e0259000. doi: 10.1371/journal.pone.0259000 (PMC8635366; doi:10.1371/journal.pone.0259000)
Supplement: Attachment — Submitted filename: Response to Reviewers.pdf [file pone.0259000.s002.pdf]

Dear Dr. Stark,

Thank you for the opportunity for us to submit a revised manuscript “Effects of health education on adolescents' non-cognitive skills, life satisfaction and aspirations, and health-related quality of life: A cluster-randomized controlled trial in Vietnam” to PLOS ONE. We are grateful for the valuable feedback and insightful comments provided by both the editor and reviewers. Most of the suggestions were incorporated in the manuscript, and we are submitting a marked-up copy of our manuscript along with the clean draft without tracked changes. We have added our responses to the editor’s and the reviewers’ comments in blue. All row numbers refer to the revised manuscript without tracked changes.

Editor’s Comments to the Authors

1. Please ensure that your manuscript meets PLOS ONE's style requirements, including those for file naming. The PLOS ONE style templates can be found at

https://journals.plos.org/plosone/s/file?id=wjVg/PLOSOne_formatting_sample_main_body.pdf

and

https://journals.plos.org/plosone/s/file?id=ba62/PLOSOne_formatting_sample_title_authors_affiliations.pdf

Author response: As suggested by the editor, we have revised the style requirement.

2. Please improve statistical reporting and refer to p-values as "p<.001" instead of "p=.000". Our statistical reporting guidelines are available at https://journals.plos.org/plosone/s/submission-guidelines#loc-statistical-reporting.

Author response: Thank you for pointing this out. It has been corrected.

3. We note that Figure 1 in your submission contain map images which may be copyrighted. All PLOS content is published under the Creative Commons Attribution License (CC BY 4.0), which means that the manuscript, images, and Supporting Information files will be freely available online, and any third party is permitted to access, download, copy, distribute, and use these materials in any way, even commercially, with proper attribution. For these reasons, we cannot publish previously copyrighted maps or satellite images created using proprietary data, such

as Google software (Google Maps, Street View, and Earth). For more information, see our copyright guidelines: http://journals.plos.org/plosone/s/licenses-and-copyright. We require you to either (1) present written permission from the copyright holder to publish these figures specifically under the CC BY 4.0 license, or (2) remove the figures from your submission:

a. You may seek permission from the original copyright holder of Figure 1 to publish the content specifically under the CC BY 4.0 license.

We recommend that you contact the original copyright holder with the Content Permission Form (http://journals.plos.org/plosone/s/file?id=7c09/content-permission-form.pdf) and the following text:

“I request permission for the open-access journal PLOS ONE to publish XXX under the Creative Commons Attribution License (CCAL) CC BY 4.0 (http://creativecommons.org/licenses/by/4.0/). Please be aware that this license allows unrestricted use and distribution, even commercially, by third parties. Please reply and provide explicit written permission to publish XXX under a CC BY license and complete the attached form.”

Please upload the completed Content Permission Form or other proof of granted permissions as an "Other" file with your submission.

In the figure caption of the copyrighted figure, please include the following text: “Reprinted from [ref] under a CC BY license, with permission from [name of publisher], original copyright [original copyright year].”

b. If you are unable to obtain permission from the original copyright holder to publish these figures under the CC BY 4.0 license or if the copyright holder’s requirements are incompatible with the CC BY 4.0 license, please either i) remove the figure or ii) supply a replacement figure that complies with the CC BY 4.0 license. Please check copyright information on all replacement figures and update the figure caption with source information. If applicable, please specify in the figure caption text when a figure is similar but not identical to the original image and is therefore for illustrative purposes only.

The following resources for replacing copyrighted map figures may be helpful:
USGS National Map Viewer (public domain): http://viewer.nationalmap.gov/viewer/

The Gateway to Astronaut Photography of Earth (public domain): http://eol.jsc.nasa.gov/sseop/clickmap/

Maps at the CIA (public domain): https://www.cia.gov/library/publications/the-world-factbook/index.html and https://www.cia.gov/library/publications/cia-maps-publications/index.html

NASA Earth Observatory (public domain): http://earthobservatory.nasa.gov/
Landsat: http://landsat.visibleearth.nasa.gov/

USGS EROS (Earth Resources Observatory and Science (EROS) Center) (public domain): http://eros.usgs.gov/#

Natural Earth (public domain): http://www.naturalearthdata.com/

Author response: We have replaced the map and obtained the Content Permission Form.

4. Please review your reference list to ensure that it is complete and correct. If you have cited papers that have been retracted, please include the rationale for doing so in the manuscript text, or remove these references and replace them with relevant current references. Any changes to the reference list should be mentioned in the rebuttal letter that accompanies your revised manuscript. If you need to cite a retracted article, indicate the article's retracted status in the References list and also include a citation and full reference for the retraction notice.

Author response: We have reviewed and revised the reference list accordingly. An additional list of references is added to the existing list to incorporate Reviewer 2's suggestions. Also, we have retracted the following two references by new ones because former ones are no longer available on the websites. *Water and Sanitation (UNICEF, 2004)* and *By the numbers: the public costs of teen childbearing (Hoffman SD, 2006)* were replaced by *Water, sanitation and hygiene interventions to combat childhood diarrhoea in developing countries (Waddington H et. al., 2009)* and *Public costs from unintended pregnancies and the role of public insurance programs in paying for pregnancy-related care (Sonfield A and Kost K, 2015)*, respectively.

Reviewers' Comments to the Authors

Reviewer #1: As the statistician reviewing this paper, I have read the paper and I recommend it to be accepted. I have no questions in regards to the data analysis as the analyses were performed rigorously and properly written up in detail for the manuscript.

Author response: Thank you!

Editor's Comments to the Authors

Reviewer #2: To the authors of this paper, I first want to commend you for writing a very clear and straightforward paper, and making a new contribution to the field of evaluating adolescent education programs for health behavior change. The paper is well written and adds a nice contribution to the literature by asking: if these programs don't achieve their primary goals (of short-term behavior change), what do they accomplish? I find that a good question to explore.

Author response: Thank you!

Overall, I think the paper could be greatly strengthened by contextualizing the research question, location of research, constraints faced by the population, and by making clearer ties to the program theory of change. I will elaborate on these points below:

The introduction is well researched and synthesizes lots of ways that negative health behaviors can have later health consequences as well as the potential costs to society. My comment on this is for the authors to consider the tone a bit in some of the writing; it currently reads as blaming adolescents for these types of societal outcomes where adolescents rarely have full control of their decisions. So much of their lives is shaped by policy, access (financial, mobility, time, permission), families, schools, wealth/resources, location of living, etc. It would strengthen this paper to reflect on how members of these age groups don't always have control, so these programs seek to shift behaviors where it seems that teenagers do have some control. The nuance would help move the focus away from adolescences' lack of interest in being more healthful, but to unpacking why they may carry certain attitudes and behaviors.

This unpacking could also lead to thinking about the programmatic theory of change – what can these programs accomplish if focusing only on education of the adolescents? Where are there barriers and constraints?

Author response: Thank you for pointing this out. As mentioned by the reviewer, it is very important not to blame adolescents assuming that it is solely adolescents' responsibility, given that there exist circumstantial factors, barriers, and constraints under which they make a health decision. To incorporate the reviewer's suggestion, we have added a paragraph elaborating on such exogenous factors that teenagers face from Row 17-29.

In the introduction, the paper would be strengthened by providing information about the specific population being studied in this trial. What are the national or sub-national statistics for these age groups on the knowledge/attitudes/behaviors that the programs seek to modify? In providing this information, it would help explain why this intervention is important (or an intervention that successfully leads to behavioral changes is important). For example, what are the rates of smoking for these age groups? What % of adolescent population starts sex at the age of this study population? How serious are these problems in the population tested?

Author response: Thank you for the suggestion. We have added statistics on risky behaviors of Vietnamese adolescents (e.g., insufficient outdoor activities) as well as consequences of such behaviors (e.g., myopia, unsafe abortions) in Row 20-29 to explain why the health education program is necessary. Please note that we reported statistics consequences associated with risky behaviors if statistics on the behaviors per se do not reflect what is happening to teens or the necessity of intervention. For example, the reported sexual intercourse prevalence among Vietnamese adolescents under 15 is very low, partly because of under-reporting attributed to social desirability bias and data collection issues. Rather than proving the prevalence of early onset of sexual intercourse for adolescents aged 11-14, we present its potential consequence, unsafe abortions, to show readers what happened in the absence of an intervention. We also presented the baseline prevalence of risky health behaviors in Table 2.

It would be additionally useful to see this information by age as adolescence is such a dynamic period of time - 11 year olds are very different from 14 year olds on many of the behaviors potentially targeted in the program.

In addition, as part of the introduction, beyond painting a picture of the scope of problems for this population in this country/region, it would be helpful to understand if there's national or subnational commitment or interest in this set of problems around adolescent unsafe or unhealthy behaviors? Are there government commitments or goals in their plans? (or global ones?) Do particular schools see this as a large issue?

Author response: Thanks for pointing this out. Although we appreciated the reviewer's feedback, we are worried that including too much information about risky behaviors may distract readers from the primary focus of this study—psychological factors. As suggested by the reviewer, it is very important to let readers know why a health program is necessary for Vietnamese adolescents by providing statistics on risky behaviors because the primary goal of a such intervention is behavior changes. Hence, we report the treatment effects of the program on health practices in the manuscript. However, we tried to limit the scope of the study to psychological factors by minimizing information on risky health behaviors per se. Given multiple topics of health education students received, we think that illustrating statistics in detail by age group and government policies on five health areas may distract readers.

In describing the program, it would be helpful for the reader if the authors can specify the programmatic goals. What are the specific health behaviors that the program is aiming to reduce/change? What are the actual program objectives and theory of change set out at the beginning of implementation? I recognize that this paper is about looking at the secondary program objectives (eg not health behavior change) but the context of what the program seeks to achieve is important in evaluating its success and whether it should be modified, stopped, scaled up, etc.

Author response: Thank you for pointing this out. As suggested by the reviewer, specific health behaviors that the program was designed to tackle are added in Row 161-165, followed by theory of change set out at the beginning in Row 165-173.

Within that program description, this would be a good moment in the paper to bring in the proposed theory of change between the primary indicators of interest for this study and the program objectives. For example, do the authors think that the indicators being studied (eg self efficacy) exist on the pathway to health behavior change ultimately? Do the programs seek to improve the measured indicators of this study or are these more unintended positive consequences? And then, making a connection between the problem statement at the intro around adolescent unhealthy behaviors and the outcomes being studied here. Why does it matter that these outcomes may improve?

Author response: Thank you for pointing this out. Accordingly, we described the proposed theory of change and how psychological factors that we evaluate are affected by the treatment in Row 165-173.

row 183 – As described, the aspirations measure seems to be more like a measure of predicted future, which feels a bit different than aspirations. For example, an adolescent may have a dream to be a doctor but feel too constrained in their

environment/lack of resources/mental health (eg depression)/etc to actually believe they can achieve becoming a doctor, so they won't share that there 5 yr vision includes being a doctor. I wonder if it's possible to explain a bit more whether this is aspirations (their hopes) or their realistic prediction of the future.

Author response: We renamed the term as aspirations gap since it is not just a measure of predicted future but a measure of the gap between future and current levels of subjective wellbeing. We define the aspirations gap as a difference between one's current and future life evaluation, says subjective general satisfaction. We assumed that this measures one's level of aspirations for better future life in general, not specific to future jobs or achievements, compared to the current level. The larger gap means that the respondent has greater hope for relatively better life while the smaller gap indicates that the one expects similar level of wellbeing in the future compared to the current level.

I am not an expert to speak to the statistical analysis method employed, so this is a question, not a suggestion. I am just wondering if analysis at the individual level should have some cluster-level coefficients (like ICCs) in the analytical model given each school constitutes a cluster of respondents?

Author response: We clustered the standard errors at the school level—the level of treatment—to take into account potential issues attributed to the clustering. As a result, our findings are robust to intraclass correlation.

Results: The paper would be helped by seeing table on baseline prevalence / rates of behaviors for key measures (primary & secondary outcomes). It's challenging to interpret results without knowing where they started. For example, are there no statistically significant changes in SRH or smoking behaviors because there's little activity or little smoking going on overall? Perhaps there just isn't much negative behavior to be reduced, so there wouldn't be a seen reduction?

Author response: Thank you for pointing this out. We have added a table reporting balance test results for dependent variables (Table 2 on page 16) in the manuscript so that readers can see the baseline values of key measures. Also, the mean and standard deviation of each dependent variable from the control group are included in Table 2 for psychological outcomes.

The paper would also be strengthened in a table that provides the measures used. For example, # of questions that measure what specifically? What behavior in what time frame? How were N/A accounted for? (for example, questions about safe sex behaviors for people not having sex).

Author response: Thank you for pointing this out. We have added a range of values in Tables 1 and 2. Please note that when analyzing the treatment effects, we report normalized effects by standardizing continuous variables by the means and standard deviations of the control group values of corresponding variables measured at baseline. Binary variables were not normalized. For sensitive questions such as sexual intercourse and smoking, students had an option *'I do not know,'* which was coded as missing. We have added this in the manuscript in Row 217-219.

Results: Is it possible in this data to disaggregate by gender and/or age group? That could be a really helpful analysis of whether girls and boys have very different or similar stats at baseline, and if changes differ by gender, as well as by age group. That could help answer if this type of programming is effective for any sub-groups within the study population. (To this point, the intro statistics on these outcomes of interest at the nationa/sub-national level would also be more interesting if broken down by age and gender).

Author response: We think that this is an excellent suggestion. We have added our heterogeneous treatment effects analysis in Row 338-355 along with Table 4. Please note that we used a continuous age variable rather than age group dummies to report the marginal treatment effects associated with one unit increase in age.

row 323/330 – The authors mention circumstantial factors - that is a really important point and I would suggest delving into that more. What could be prohibiting use of behaviors? This would be very worthwhile exploration in the discussion section – what are the barriers to the behavior change that are not just individual motivation? Access? Policy? Time? Resource? As an example, is there limited option for exercise because students are so busy with schoolwork and help around the house and supporting with taking care of their siblings? I think that exploration could then connect to reflections on what kind of intervention COULD lead to the program objective of behavior change on key behaviors.

Author response: We agree that this is a very important point. In the revised manuscript, we delved into potential mechanisms through which a health education program did not lead to behavior changes of adolescents, including circumstantial factors in Row 367-403.

Last, on this topic of limitations of the intervention design, this reviewer finds that the authors could write a bit more that directly acknowledges that the intervention as designed doesn't seem to accomplish its primary goal (of changing behaviors). What is in the literature that does led to reductions in smoking commencement, early sexual debut, unprotected sex, etc? Is it about teaching methods? Or frequency/duration of

lessons? Perhaps who the teachers are really matters? Could those be incorporated into this type of intervention to make a difference? Otherwise, I think the authors should be frank in the final discussion that perhaps the intervention cannot achieve its stated program objectives – so either a different intervention design is needed, or else people who want to implement these programs need to understand that the programs can achieve interesting outcomes related to self esteem, etc, as shown in this analysis, but the expectation should not be on behavior change.

Author response: We agree that it is important to acknowledge the fact that the intervention did not achieve its primary goal of behavior changes. We have added the following in Row 361-363: *"However, the program had limited effects on reducing risky health behaviors—the primary objective of the information-based intervention."* Besides, we have proposed three potential mechanisms through which we did not find behavior changes despite the increased self-efficacy in Row 367-403.

Finally, in the discussion, it is worth talking about how the typical assumption of knowledge --> changed attitudes --> changed behaviors is really challenged by the evaluations of these interventions. Perhaps the authors could talk about how the theory of change needs to be re-evaluated because it clearly isn't so linear to get results. There's a robust literature out there on how this assumed linear model does not show itself to be true across many programs with information provision as the primary activity, it would be great to delve into that more.

Author response: Thank you for pointing out the necessity of revisiting the theory should be re-evaluated. Although we agree that this is an important task to do, our findings do not allow us to through which mechanisms health education failed to cause behavior changes among adolescents. We have proposed three potential mechanisms through which the program was unable to achieve its goal of behavior changes: absence of students' intentions; the lack of room for students to alter their health behaviors; and the timeframe of data collection. Hence, further study is necessary for us to argue the theory of change should be re-revaluated in a certain way. Besides, we tried to focus on the causal effects of health education on psychological factors that are often neglected when evaluating the information-based approach rather than mechanisms through which health education fails in behavior changes.

row 375 – The authors mention that the time period is shorter than planned due to the COVID-19 pandemic. It would be helpful to lay that out earlier in methods - I was definitely curious why the 5 month follow up period, and having that it was planned for longer but had to be modified is helpful context for reading further.

Author response: As suggested, the delay in data collection is noted under Procedure in Row 198-200.

And then I have a few very small comments from the results section:

row 221 - specify the significance level instead of saying “at any conventional levels”. - 5% 10%?

Author response: Thank you for pointing this out. We have revised it in Row 250 as follows: *“they were not statistically significant at the 5 percent level.”*

row 264 - is that 0.067 SDs difference or scores difference? Text says scores but sentence follows format of others which list SDs, so just want to clarify.

Author response: Thank you for pointing this out. It is SDs as you said, and we have corrected it accordingly in Row 298.

row 280 - say stat insignificant instead of “estimated with large standard errors” if that’s what you mean.

Author response: Thank you for pointing this out. We have revised it in Row 313-314 as follows: *“the differences are not statistically significant”*.

Thank you for writing a paper on this interesting topic and taking a unique angle to studying these interventions by asking what non-primary outcomes are achieved in these programs. With these modifications, I think this will be a strong contribution to the conversation.

Author response: Thank you!
